# Conceptualisation of health among young people: a protocol for systematic review and thematic synthesis of qualitative studies

**DOI:** 10.1186/s13643-024-02614-0

**Published:** 2024-07-27

**Authors:** Katrin Metsis, Joanna Inchley, Andrew James Williams, Sebastian Vrahimis, Lamorna Brown, Frank Sullivan

**Affiliations:** 1 School of Medicine, University of St Andrews, North Haugh, St Andrews, KY16 9TF UK; 2 MRC/CSO Social and Public Health Sciences Unit, School of Health and Wellbeing, University of Glasgow, Clarice Pears Building, 90 Byres Road, Glasgow, G12 8TB UK

**Keywords:** Self-reported health, Young people, Adolescent, Concept formation, Qualitative research, Self report, Inequalities

## Abstract

**Background:**

Self-reported health is a widely used health indicator in surveys and questionnaires. The measure gained attention when research identified its association with mortality in the 1970s and 1980s. The measure is also associated with morbidity and other health outcomes such as the utilisation of health services. Self-reported health is a particularly useful measure for young people because this age group is generally clinically healthy. However, it is known that many chronic conditions have long latency periods that are initiated early in life. Because of its predictive nature, self-reported health can be used to estimate young people’s current and future health. Despite its widespread use, however, self-reported health remains a poorly understood concept. This paper presents the protocol for a systematic review that will identify and synthesise qualitative studies that investigate the factors that are considered by young people when they assess their health, and when they talk about health overall.

**Methods:**

The population of the review is young people aged 10–24 years, with or without health conditions. We will search the databases of MEDLINE (Ovid®), PsycINFO (APA PsycNet), ProQuest Sociology Collection, and Web of Science Core Collection™. We will also utilise techniques of reference checking and forward citation searching, as this strategy has been shown to result in a higher number of high-quality studies in social science systematic reviews. Google Scholar and Google Search were used during preliminary searches; Google Scholar will be utilised for forward citation searching. We will include studies written in English, German, or Finnish; there will be no lower date limit. One reviewer will screen all citations. A second reviewer will independently screen a sample of 20% of the abstracts. Data will be extracted by one researcher, two other researchers will independently review all data extracted, and quality appraisal will be completed by the first reviewer. We will utilise the Quality Framework for the appraisal of included articles and thematic synthesis of qualitative studies.

**Discussion:**

The results of this systematic review will improve the understanding of the factors that are considered during the self-assessments of health; this will improve the interpretation of the results of quantitative research. Also, an improved understanding of the conceptualisation of health will inform the development of health policies and interventions that support young people’s health.

**Systematic review registration:**

PROSPERO CRD42022367519.

**Supplementary Information:**

The online version contains supplementary material available at 10.1186/s13643-024-02614-0.

## Background

Self-reported health (SRH) is a widely used health indicator in surveys and questionnaires. Questions such as ‘How is your health in general?’ followed by response options of ‘Very good’, ‘Good’, ‘Fair’, ‘Bad’, or ‘Very bad’ are included in many questionnaires. The SRH measure gained attention when research identified its association with mortality in the 1970s and 1980s [1]. Later, different studies have demonstrated that SRH is associated with functional health, treatment outcomes, biomarkers, and utilisation of health services in different population groups, including young people [2–5]. The measure is included in national and international surveys such as the Scottish Health Survey and the European Union Statistics on Income and Living Conditions (EU-SILC). SRH measure is useful for studying young people’s health for several reasons. First, children and adolescents do not often present objective clinical symptoms; however, SRH starts to decline during early adolescence due to subjective complaints [6]. It is known that many chronic conditions such as cardiovascular disease or cancers have long latency periods; the World Health Organization (WHO) has estimated that 70% of premature deaths among adults are primarily due to behaviours initiated during adolescence [7]. Lynch and Smith [8] have shown that many risk factors of coronary heart disease, type 2 diabetes, or chronic obstructive pulmonary disease are present across life course stages, including adolescence. Because clinical endpoints are not common in adolescence and young adulthood, SRH is an appropriate measure to assess health [9–11].

Despite its widespread use and association with objective health outcomes, however, SRH remains a poorly understood measure [1]. Research has found that the main aspects which impact self-ratings of health among adults are physical health problems, functional capacities, health behaviours, and psychological aspects [12]. Studies focusing on young people have found that rating their health is based on social, mental, and physical aspects such as lifestyle, possessions and space, perceived stressors, social belonging, medical conditions, and physical appearance [13]. These different factors are captured in the definition of SRH as suggested by Tissue [14]:

‘…it represents a summary statement about the way in which numerous aspects of health, both subjective and objective, are combined within the perceptual framework of the individual respondent.’ (p.93).

Jylhä [1] has concluded that the rating of a person’s health arises from the cognitive reasoning process where people evaluate information about their conditions and sensations.

Numerous definitions of health capture different dimensions of health. As Larson [15] has pointed out, that is also the reason why there is no agreement on the meaning of health—health is a complex phenomenon that includes medical, social, economic, and other components. The most prominent definition, the WHO definition of health that is part of its constitution [16], describes health as:

‘A state of complete physical, mental and social well-being and not merely the absence of disease or infirmity.’ (p.1).

The Constitution of the WHO [16] also asserts that the highest attainable standard of health is a fundamental right of every human being and fundamental to the attainment of peace and security. The WHO definition of health was expanded on by the Ottawa Charter for Health Promotion [17]:

‘to reach a state of complete physical mental and social wellbeing, an individual or group must be able to identify and to realize aspirations, to satisfy needs, and to change or cope with the environment. Health is, therefore, seen as a resource for everyday life, not the objective of living.’ (p.1).

Larson [15] has categorised the WHO definition of health as a separate conceptual model of health due to its prominence and comprehensive nature. Larson [15] presents three further models of health: medical, wellness, and environmental models of health. The medical model refers to the absence of disease and disability. The wellness model has an emphasis on the link between mind and body and stresses that health is more than the absence of illness incorporating positive dimensions of well-being, energy, and ability to work. The environmental model places individuals within physical, social, and other environments and emphasises their ability to maintain a healthy balance. However, Larson [15] suggests that models simplify health and invites one to reflect on the complexity, as even a health assessment based on all four models would be a simplification of reality. Given the plethora of different definitions of health that incorporate different dimensions, McCartney et al. [18] reviewed the definitions of health and suggested that, for public health, the best definition should incorporate different dimensions of health and apply to individuals and populations. McCartney et al. [18] suggested the adoption of the definition of health as provided by Last in the dictionary of public health [19]:

‘A structural, functional and emotional state that is compatible with effective life as an individual and as a member of society.’

Given the complexity of the concept of health, it is important to understand how the term is understood within a research context. This paper presents the protocol of a systematic review of qualitative studies that investigate how young people reason during their self-assessments of health and what factors they include when discussing the meaning of ‘health’. The systematic review is part of a PhD project that investigates health inequalities among Scottish young people and uses data from the UK Censuses and Scottish Longitudinal Study. These data sources include a general health question that is used to operationalise health. The systematic review will complement quantitative analysis by synthesising the themes and factors that have been identified by young people when they discuss the meaning of health.

## Methods

This systematic review will address the following questions.

1. How do young people reason when they answer self-reported health questions in the surveys?
2. How do young people reason when they rate health as very good, good, fair, or bad?
3. How do young people understand the concept of health generally?

The review protocol was registered in the PROSPERO, registration number is CRD42022367519. The guidelines of the Preferred Reporting Items for Systematic Reviews and Meta-Analyses Protocols (PRISMA-P) [20] are followed in reporting this protocol; a completed checklist is provided as an additional file (Additional file 1). The findings of the systematic review will be reported following the Enhancing Transparency in Reporting the Synthesis of Qualitative Research (ENTREQ) statement [21].

PICOS framework [22] was used to formulate the research question and preliminary search strategy.

### Participants and eligibility criteria

The study population is young people aged 10–24 years, with or without health conditions. The term ‘young people’ covers two life stages. The WHO [23] defines ‘adolescence’ as a life stage between 10 and 19 years. Second, people aged 15–24 are defined as ‘youth’. People aged 10–24 are defined as ‘young people’. This grouping is visualised in Table 1.

**Table 1:** Study population

Adolescents: aged 10–19	Youth: aged 15–24
Young people: aged 10–24 years	

The outcome of this review will be the synthesis of factors that are discussed by young people when they explore the concept of health. To achieve this outcome, we will locate and synthesise qualitative primary studies. We will apply the definition of qualitative research as proposed by Aspers and Corte [24]:

‘iterative process in which improved understanding to the scientific community is achieved by making new significant distinctions resulting from getting closer to the phenomenon studied.’ (p. 155).

Studies using the data collection methods listed below will be included.

1. Different types of interviews (individual semi-structured or in-depth interviews, focus groups, or group discussions)
2. Visual methods such as photography, drawings, or mind maps
3. Written accounts that describe photographs
4. Data from open-ended survey questions under the condition that data were analysed by using qualitative methods such as content analysis

Studies using the word ‘health’ in the research question(s) will be included. Studies using the word ‘feel’ will also be included if the study aims to investigate the perception of health. Some studies have used the word ‘feel’ when exploring the conceptualisation of health because of cultural reasons. For example, as Joffer et al. [25] explain, in Sweden, ‘feel’ is often used when asking about one’s health. Spencer [26] has explored the meaning of health by using the word ‘feel’, which captures the holistic conceptualisation of health. We decided to exclude related terms of wellbeing, quality of life, and health-related quality of life. This systematic review aims to inform (survey) research that utilises SRH measure. Therefore, we will focus on studies that explored the concept of health by asking young people explicitly about its meaning and used the term ‘health’. Also, there is evidence that, compared to the SRH, these are perceived as different constructs [27, 28].

The exclusion criteria are as follows.

1. Studies exploring the concept of wellbeing, quality of life, or health-related quality of life will be excluded
2. Studies that investigated the conceptualisation of health in the context of certain phenomena such as healthy eating will be excluded because this explores the concept of health from pre-defined aspects
3. Papers without any empirical aspect will be excluded
4. Conference abstracts will be excluded
5. Editorials, opinion pieces, and book reviews will be excluded
6. Studies are limited to those written in English, German, or Finnish
7. There is no lower date limit for publications

Visual methods such as photovoice [29] are known to add details to interviewees’ perceptions of phenomena and allow participants to express their ideas in a non-verbal way. There is no consensus if the open-ended questions in the surveys are qualitative or quantitative data [30]. Compared to the interview data, written data can result in less depth as respondents’ statements cannot be further explored [31]. However, open-ended questions can also be seen as descriptions of phenomena with concepts used by ordinary citizens [32]. These descriptions can be analysed by using qualitative methods, for example, content analysis allows for developing categories that increase the understanding of the phenomenon. Because one of the aims of this review is to understand the concept of health in the survey context, we felt that written accounts, if analysed as qualitative data, will contribute to the understanding of the phenomenon. We will consider the impact of different data collection methods and types of data during the synthesis of the studies. Inclusion and exclusion criteria by the PICOS framework [22] are summarised in Table 2.

**Table 2:** Inclusion and exclusion criteria by the PICOS framework

	Inclusion criteria	Exclusion criteria
Population	10–24-year-old respondents with or without health conditions	The study includes participants from a wider age range, and it is not possible to separate 10–24-year-olds
Intervention	Studies that investigate how young people conceptualise health (1) in a survey context and (2) overall The study question includes the term ‘health’ or ‘feel’	Concepts of wellbeing, quality of life, health-related quality of life
Comparison	Not applicable	Not applicable
Outcome	‘Conceptualisation of health’: synthesis of factors discussed by young people when they discuss the concept of health	Not reporting outcomes of interest. Insufficient detail for data synthesis Studies that investigated the conceptualisation of health in the context of certain phenomena such as physical activity or diet
Study type	Qualitative studies Visual methods such as photography, mind maps, drawings, and written descriptions of drawings or photographs Open-ended survey questions that have been analysed by qualitative methods Mixed methods studies if it is possible to extract qualitative findings only	Quantitative studies Mixed method studies if it is not possible to separate qualitative findings Conference abstracts, opinion pieces Book reviews Studies without any empirical aspect

### Search strategy

Different authors [33–35] have drawn attention to the problems of the inclusion of qualitative evidence in systematic reviews. In this review, the identification of the keywords will be an iterative process. The search strategy will be discussed with all authors. In preparation for this review and study protocol, we used general keywords such as ‘adolesc*’, ‘health’, and ‘self-reported health’. These will be complemented with the keywords from identified relevant studies and refined during the searches. We will identify both Medical Subject Headings (MeSH) terms for the MEDLINE searches and keywords.

In preparation for the review, we have tested three tools to identify search terms. Tools such as PICO are frequently used in systematic reviews [36]. However, it is felt that his tool is not suitable for identifying qualitative studies [36]. As a response, Cooke et al. [37] developed the SPIDER tool which includes five core concepts:

1. Sample
2. Phenomenon of interest
3. Design
4. Evaluation
5. Research type

However, Methley et al. [38] found that although the SPIDER tool had a higher specificity compared to the PICO and PICOS tools [22], it omitted many relevant articles, possibly due to problems in the indexing of qualitative studies. We, therefore, compiled a preliminary search strategy for all three tools. We found that the PICO framework resulted in search terms that brought up a very large number of studies, and similarly to Methley et al. [38], the number of studies was reduced when using the SPIDER tool. Therefore, we used the PICOS framework to identify the preliminary set of search terms. This tool is also recommended by Methley et al. [38] when time and resources are limited. The PICOS mnemonic does not include the component of ‘context’ that is recommended for systematic reviews of qualitative evidence (PICO mnemonic) [39]. In this systematic review, relevant studies from all geographical regions or different population groups will be included. The search strategy for MEDLINE (Ovid) is included in Additional file 2.

We will search the following databases.

1. MEDLINE (Ovid®)
2. PsycINFO (APA PsycNet)
3. ProQuest Sociology Collection (Applied Social Sciences Index & Abstracts (ASSIA)/Sociological Abstracts/Sociology Database)
4. Web of Science Core Collection™. This collection includes databases of the Science Citation Index, Social Sciences Citation Index, Arts & Humanities Citation Index, Conference Proceedings Citation Index, Book Citation Index, Emerging Sources Citation Index, Index Chemicus, Current Chemical Reactions, Preprint Citation Index

The selection of databases was informed by the nature of the research question which stands in the intersection of medicine, sociology, psychology, and survey research. We considered the inclusion of the following databases.

- The CINAHL database was not included because of its focus on nursing
- Embase has a focus on biomedical literature, and we felt that this aspect will be represented in MEDLINE searches
- We did not include the PubMed database because it is considered to have a similar coverage to MEDLINE
- We searched the Cochrane Database of Systematic Reviews during the development of the review question

We will also use the techniques of reference checking and forward citation searching; Papaioannou et al. [35] and Greenhalgh et al. [40] have demonstrated that compared to conventional database searches, this strategy results in a higher number of high-quality studies in social science systematic reviews. Google Scholar and Google Search were used as supplementary tools during preliminary searches; Google Scholar will be utilised for forward citation searching. Studies are limited to those written in English, German, or Finnish. There is no lower date limit for publications.

### Data screening and extraction

A reference library will be created and maintained in EndNote20; the title and abstract of retrieved studies will be uploaded to the library. One researcher (KM) will search databases, upload retrieved studies to the library, and screen all titles and abstracts for the inclusion of the studies. The second researcher (SV) will randomly select and screen a sample of 20% of the abstracts to establish an inter-rater agreement. Disagreements will be resolved by discussion; where consensus cannot be reached, a third researcher (FS) will be consulted.

Data will be extracted by one researcher (KM). MS Word and Excel documents will be used to manage data extraction; the data extraction form was piloted during preliminary searches. The second and third researchers (SV and LB) will independently review all data extracted by the first reviewer (KM). KM will discuss the screening and inclusion of the studies with the review team regularly. The review team includes expertise in systematic review methodology (FS). We will report the agreement rates for data screening, extraction, and quality appraisal.

The following information will be extracted from the included studies.

1. Study reference
2. Country
3. Aim of the study
4. Sample characteristics
5. Format of the health question
6. Data collection method
7. Data analysis method
8. Key findings

### Quality appraisal

Dixon-Woods et al. [34] have recognised that the formal synthesis of qualitative research is difficult because of the underdeveloped techniques for searching, selecting, and appraisal. However, because qualitative evidence is increasingly used to inform decision-making processes, the question has arisen about whether and how the trustworthiness of qualitative evidence should be appraised [41–43]. Although several appraisal tools have been developed for qualitative research, there is no consensus on the appropriate quality criteria to evaluate the qualitative findings. It is also argued that many tools incorporate quality dimensions that characterise quantitative results [43].

The Cochrane Handbook [44] emphasises that researchers need to decide which appraisal tool is most appropriate for a particular review.

To select the appraisal tool, we have considered three instruments.

1. The Critical Appraisal Skills Programme (CASP) quality assessment tool for qualitative studies [45]
2. The Joanna Briggs Institute’s (JBIs) Critical Appraisal Checklist for Qualitative Research [46]
3. The Quality Framework (the QF) [42]

For the appraisal of included articles, the QF will be applied [42]. The JBI’s Critical Appraisal Checklist for Qualitative Research was not chosen because it has an emphasis on congruity between philosophy, methodology and methods [41, 46]. Maijd and Vanstone [41] have argued that if the appraisal framework has an emphasis on theoretical underpinnings, then descriptive studies that do not include rigorous theoretical discussion can be classified as untrustworthy. However, descriptive research can produce findings that are relevant to understanding respondent perspectives [41, 47]. We felt that the CASP qualitative checklist [45] which is easy to administer and widely used in qualitative evidence synthesis did not cover the dimensions of qualitative research as thoroughly as the QF. One potential reason, as Williams et al. [43] argued, is that this tool was developed during the 1990s alongside quantitative research tools. The QF [42] was developed as a response to the growing need to appraise qualitative evidence which is increasingly used in government evaluations. Although the framework was developed to assess qualitative evaluation across Government Departments, it is also suitable to appraise evidence from different types of qualitative inquiry such as reports or journal papers [42].

The QF includes 18 questions and a set of quality indicators for each question that can be used to guide the appraisal process. To make the appraisal results transparent, we will develop a grading system. Unlike the CASP [45] or JBI checklist [46], the QF does not have a grading system. The grading system will be based on existing grading grids and calibrated based on included studies. No studies will be excluded based on quality alone. All studies that meet inclusion criteria will be included in the review regardless of their quality score. The quality of the included studies will be assessed by the first reviewer; these results will be independently assessed by the second (SV) and third (LB) reviewers.

### Strategy for data synthesis

A thematic analysis strategy [48, 49] will be used to synthesise selected qualitative studies. This approach is recommended by the Cochrane Systematic Review Group for the synthesis of qualitative studies [36]. We will apply the adoption of the thematic synthesis as outlined by Thomas and Harden [49]. This comprises three stages: (1) coding of the findings of primary studies, (2) organising the codes into descriptive themes, and (3) developing analytical themes. The NVivo software will be used to synthesise included qualitative studies. Depending on the data, we will analyse the results by gender, age, location, or data collection method.

## Discussion

This paper outlines the protocol for the systematic review of qualitative studies that investigate how young people understand the concept of health and how they reason when rating their health. The strength of this review will be the focus on young people’s interpretation of health rather than the association between health ratings and symptoms. We will include studies completed in different contexts and among different sub-groups; the review team includes researchers with a background in nursing (KM), clinicians (SV and FS), social sciences (KM, JI, AJW), and computer science (LB). These aspects will provide insight into diverse aspects of health. The limitations of this review are the limited time and resources. Preliminary searches have demonstrated that the number of identified studies can be very large due to the breadth of search terms (over 5000). Also, because qualitative studies can be hard to identify through database searches, we anticipate that forward and backwards citation searching will be challenging. However, to the best of our knowledge, this will be the first systematic review of qualitative studies that investigate young people’s conceptualisation of health. Therefore, the results will improve the understanding of self-assessments of health and thus the interpretation of quantitative health research. Also, an improved understanding of the conceptualisation of health will inform the development of health policies and interventions that support young people’s health.

### Supplementary Information

Additional file 1. PRISMA-P checklist.

Additional file 2. MEDLINE (Ovid) search strategy.

## Data Availability

Not applicable.

## Abbreviations

EU-SILC: The European Union Statistics on Income and Living Conditions survey
CASP: The Critical Appraisal Skills Programme
SRH: Self-reported health
QF: The Quality Framework
WHO: The World Health Organization
