# Supplementary material for: Conceptualisation of health among young people: a protocol for systematic review and thematic synthesis of qualitative studies
Source: Syst Rev. 2024 Jul 27;13:200. doi: 10.1186/s13643-024-02614-0 (PMC11283722; doi:10.1186/s13643-024-02614-0)
Supplement: Supplementary file 2 — Additional file 2. MEDLINE (Ovid) search strategy. [file 13643_2024_2614_MOESM2_ESM.docx]

**Additional file 2. MEDLINE (Ovid) search strategy**

| MEDLINE (Ovid®) | | |
| --- | --- | --- |
| Population | 1 | Adolescent/ or Adolescent Health/ or Adolescent Medicine/ or Young Adult/ |
|  | 2 | (adolescen* or youth* or young or young people or young adult or teen*).mp. |
|  | 3 | 1 or 2 |
| Intervention  (SRH) | 4 | "surveys and questionnaires"/ or health surveys/ or patient health questionnaire/ or exp self report/ |
|  | 5 | (Self-assessed health or Self-rated health or Perceived health or Self-evaluated health or Self-reported health or Self-ratings of health or Self-assessments of health or Self-perceptions of health or Self-evaluations of health or Self-evaluation of health).mp. |
|  | 6 | ((subjective or self-report* or self-assess* or self-rat* or perce* or self-evaluat*) adj5 health).mp. |
|  | 7 | Health Status/ |
|  | 8 | 4 or 5 or 6 or 7 |
|  | 9 | 3 and 8 |
| Comparison | NA |  |
| Outcome  (conceptualization) | 10 | exp thinking/ or exp concept formation/ or judgment/ |
|  | 11 | exp diagnostic self evaluation/ |
|  | 12 | exp Comprehension/cl, ph [Classification, Physiology] |
|  | 13 | Attitude to Health/eh, sn [Ethnology, Statistics & Numerical Data] |
|  | 14 | ((Conceptualis* or perce* or perception or view or feel or subjective) adj5 health).mp. |
|  | 15 | 10 or 11 or 12 or 13 or 14 |
|  | 16 | 9 and 15 |
| Study type | 17 | qualitative.mp. |
|  | 18 | 16 and 17 (N=2048) |
|  | 19 | limit 18 to structured abstracts (N=1253) |
|  | 20 | limit 19 to english language (N=1218) |
